# Dysbiosis of the gut microbiota as a susceptibility factor for Kawasaki disease

**DOI:** 10.3389/fimmu.2023.1268453

**Published:** 2023-10-31

**Authors:** Yoshiki Teramoto, Shohei Akagawa, Shin-ichiro Hori, Shoji Tsuji, Koichiro Higasa, Kazunari Kaneko

**Affiliations:** ^1^ Department of Pediatrics, Kansai Medical University, Hirakata, Osaka, Japan; ^2^ Department of Genome Analysis, Institute of Biomedical Science, Kansai Medical University, Hirakata, Osaka, Japan

**Keywords:** Kawasaki disease, gut microbiota, dysbiosis, 16S rRNA sequencing, *Ruminococcus gnavus*, *blautia*

## Abstract

**Introduction:**

Gut microbial imbalance (dysbiosis) has been reported in patients with acute Kawasaki disease (KD). However, no studies have analyzed the gut microbiota while focusing on susceptibility to KD. This study aimed to evaluate whether dysbiosis elevates susceptibility to KD by assessing children with a history of KD.

**Methods:**

Fecal DNA was extracted from 26 children with a history of KD approximately 1 year prior (KD group, 12 boys; median age, 32.5 months; median time from onset, 11.5 months) and 57 age-matched healthy controls (HC group, 35 boys; median age, 36.0 months). 16S rRNA gene analysis was conducted with the Illumina Miseq instrument. Sequence reads were analyzed using QIIME2.

**Results:**

For alpha diversity, Faith’s phylogenetic diversity was significantly higher in the KD group. Regarding beta diversity, the two groups formed significantly different clusters based on Bray–Curtis dissimilarity. Comparing microbial composition at the genus level, the KD and HC groups were significantly different in the abundance of two genera with abundance over 1% after Benjamini–Hochberg false discovery rate correction for multiple comparisons. Compared with the HC group, the KD group had higher relative abundance of *Ruminococcus gnavus* group and lower relative abundance of *Blautia*.

**Discussion and conclusion:**

*Ruminococcus gnavus* group reportedly includes pro-inflammatory bacteria. In contrast, *Blautia* suppresses inflammation via butyrate production. In the predictive functional analysis, the proportion of gut microbiota involved in several pathways was lower in the KD group. Therefore, dysbiosis characterized by distinct microbial diversity and decreased abundance of *Blautia* in parallel with increased abundance of *Ruminococcus gnavus* group might be a susceptibility factor for KD.

## 1 Introduction

Kawasaki disease (KD), first reported by Tomisaku Kawasaki in 1967, is a systemic vasculitis that predominantly affects children between the ages of 6 months and 5 years. (1) The incidence of KD is 100 per 100,000 persons worldwide. (2) In Japan, the incidence is 200–300 per 100,000 persons (3).

Some antigens are thought to be the main cause of KD, but research to date has not been able to identify a specific causative antigen. Therefore, an abnormal host immune response is hypothesized to be necessary for KD to develop. In other words, KD is a multifactorial disease in which either genetically or environmentally susceptible individuals develop the disease through an excessive immune response triggered by infection. (4) Cesarean delivery, (5) formula feeding, (6) and history of antimicrobial use prior to the onset of KD (5) have been reported to be environmental risk factors for the development of the disease. These factors also have a significant impact on the intestinal microbiota, which leads to the hypothesis that disturbances in the gut microbiota, i.e., dysbiosis, might increase susceptibility to KD.

There have been three previous reports on the dysbiosis in patients with KD. (7–9) However, all studies have included patients in the acute phase of KD. One study reported transient changes in intestinal microbiota during the elevation of body temperature, which is common with viral infections. (10, 11) As a result, it is difficult to determine whether dysbiosis in the acute phase of KD is the result of KD or the factor that led to KD onset. In this study, we aim to clarify whether dysbiosis is a susceptibility factor for KD by comparing the intestinal microbiota of children with a history of KD with that of healthy children in the same age range.

## 2 Materials and methods

### 2.1 Ethics approval and consent

This study was performed at Kansai Medical University Hospital (Osaka, Japan) and Osaka Asahi Children’s Hospital (Osaka, Japan). The study protocol was approved by the ethics committees of Kansai Medical University (approval no. 2015127) and Osaka Asahi Children’s Hospital (approval no. 43). Written informed consent was obtained from the parents of all participants before enrolment.

### 2.2 Participants and study design

This case-control study was conducted between March 2017 and December 2021. Participants consisted of 26 pediatric outpatients aged 15–64 months at the time of sample collection who had been diagnosed with KD within the latest 6–15 months and 57 healthy age-matched controls without a history of KD.

The diagnosis of KD was based on diagnostic guidelines published by the Japanese Society of Kawasaki Disease. (1) A patient was considered to have KD if they have five or more of the following principal features, or four features with other diseases ruled out: 1) fever; 2) bilateral bulbar conjunctival injection; 3) redness of the lips, strawberry tongue, or erythema of the oral and pharyngeal mucosa; 4) rash; 5) erythema and edema of the hands and feet; and 6) cervical lymphadenopathy. Previous history of KD, presence of coronary artery aneurysms, and responsiveness to treatment were not considered. Exclusion criteria consisted of antibiotic use within 1 month of sample collection, a diet with calorie or macronutrient restriction, history of hospitalization or surgery except for KD, regular visits to the hospital for other medical reasons, and current medication use. Individuals recruited as healthy controls (HCs) were from the same age group, lived in the same area, had no history of hospitalization within 1 year of sample collection, and had no underlying medical conditions such as allergic diseases or a history of KD. The primary outcome was the difference in the relative abundance of a genus in the gut microbiota between the two groups. The secondary outcomes were alpha and beta diversity and predicted function of the microbiota.

Initial treatment consisted of either IVIG only or IVIG plus steroids; the selection was based on scores on several risk scales for IVIG resistance (12–16). If the patient did not respond to the initial treatment, additional IVIG was administered. If the patient did not respond to the second IVIG treatment, infliximab was chosen as third-line treatment. Aspirin was administered to all patients from the beginning of treatment to 1 month after the last treatment. After hospital discharge, patients were monitored for coronary aneurysms with echocardiography in an outpatient clinic. Patients without aneurysms did not receive further treatment.

### 2.3 Sample collection and DNA extraction

Using a stool sampling kit (TechnoSuruga Laboratory, Shizuoka, Japan), 0.5 g of spontaneously defecated stool were collected. The sampling kit contains guanidine thiocyanate, which enables the sample to be stored at room temperature. The samples were sent to our institution. DNA was immediately extracted using NucleoSpin DNA Stool (MACHEREY-NAGEL, Dueren, Germany). The extracted DNA was stored at −80°C until further analysis.

Blood testing was only performed during the acute phase of KD. At the time of fecal sampling, all participants in the KD group were completely healthy with no clinical symptoms or signs suggesting KD. Therefore, blood sampling was not performed.

### 2.4 16S rRNA gene sequencing

16S rRNA gene analysis was performed at Macrogen Japan (Tokyo, Japan). Library preparation was based on Illumina 16S Metagenomic Sequencing Library protocols for the V3 and V4 regions. Polymerase chain reaction (PCR) was conducted using genomic DNA 2 ng, 5X reaction buffer, IMm dNTP mix, 500 nM universal F/R PCR primer, and Heraculase II fusion DNA polymerase (Agilent Technologies, Santa Clara, CA, USA). Adapter overhang sequences were as follows: V3-F: 5′-TCGTCGGCAGCGTCAGATGTGTATAAGAGACAGCCTACGGGNGGCWGCAG-3′ and V4-R: 5′-GTCTCGTGGGCTCGGAGATGTGTATAAGAGACAGGACTACHVGGGTATCTAATCC-3′. The first PCR was performed at 98°C for 3 minutes of thermal denaturation, followed by 25 cycles of 95°C for 30 seconds, 55°C for 30 seconds, and 72°C for 30 seconds, with a final extension step at 72°C for 5 minutes. After the first PCR, the product was purified using AMPure (Agencourt Bioscience, Beverly, MA, USA) to remove the primer. In the second PCR, 2 µL of the first PCR product was subjected to PCR using the Illumina Nextera XT Index kit (Illumina, San Diego, CA, USA). The conditions for the second PCR were the same as for the first PCR, with 10 cycles. After the second PCR, the product was purified using AMPure. The concentration of the PCR product was measured using quantitative PCR (qPCR) with the qPCR Quantification Protocol Guide (KAPA Library Quantification kits for Illumina Sequencing platforms). Qualitative confirmation was carried out with TapeStation D1000 ScreenTapes (Agilent Technologies, Waldbronn, Germany). The generated libraries were sequenced on the Illumina Miseq instrument (Illumina, San Diego, CA, USA).

Sequence reads were imported into QIIME2 and analyzed for bacterial identification and diversity. (17) Paired-end FASTQ files underwent Phred quality score-based quality filtering, paired-end merging, chimera removal, singleton removal, and feature table construction consisting of amplicon sequence variants. Alpha diversity was calculated with observed species, Shannon Index, Simpson Index, and Faith’s phylogenetic diversity. Beta diversity was calculated with Bray–Curtis dissimilarity. The PERMANOVA test was used for comparisons between the two study groups. To compare the bacterial compositions of the groups, relative abundance at the phylum and genus levels of the two groups was analyzed. Prediction of functional profiles from the 16S rRNA dataset was performed using Phylogenetic Investigation of Communities by Reconstruction of Unobserved States 2 (PICRUSt2) software and the Kyoto Encyclopedia of Genes and Genomes (KEGG) database (release 70.0) according to the tutorial on the official website. (18) Between-group differences in bacterial genus abundance or KEGG pathways were analyzed with Welch’s t-test with false detection rate correction using STAMP software (19).

### 2.5 Statistical analysis

Continuous variables are represented as medians and IQRs. Categorical variables are represented as numbers and percentages. The Chi-squared and Mann–Whitney U tests were used for comparisons between the two study groups. Statistical significance was set at p < 0.05. For comparing relative abundance of bacterial taxa, Benjamini–Hochberg false discovery rate (FDR) correction was performed for multiple comparisons. FDR q-value < 0.10 was considered significant (20).

To calculate the appropriate sample size for the study, we conducted *a priori* power analysis using G*Power version 3.1.9.4 (Heinrich Heine University, Düsseldorf, Germany), (21) with an effect size of 0.6 and a type I error rate of 0.05. Since sample sizes of 28 for the KD group and 56 for the HC groups would provide a power of 0.8, we set the target number of participants to be 28 and 56 for the groups. To verify the results, a *post hoc* power analysis for the relative abundance of *Blautia* was performed with a type I error rate of 0.05. All other statistical analyses were performed with JMP Pro 16 (SAS Institute Japan, Tokyo, Japan).

## 3 Results

### 3.1 Patient characteristics

The KD group included 26 patients (12 boys). The HC group included 57 children (35 boys). Median age at sample collection was 32.5 months (interquartile range (IQR), 25.0–45.2 months) for the KD group and 36.0 months (IQR, 24.5–48.0 months) for the HC group. There were no significant differences in age and type of nutrition, such as breast milk or formula feeding, between the groups (p=0.50 and p=0.18, respectively). Meanwhile, in terms of mode of delivery, Cesarean delivery was more common in patients with KD than in HCs, although this difference was not statistically significant (p=0.054). In the KD group, the median duration between KD onset and sample collection was 11.5 months (IQR, 10.7–13.0 months) (**Table 1**). Clinical characteristics and laboratory indexes for KD are shown in **Table 2**.

**Table 1:** General characteristics of the study participants.

	KD group (n=26)	HC group (n=57)	p-value
Male sex [n (%)]	12 (46%)	35 (61%)	0.19
Median age at sample collection, months (interquartile range)	32.5 (25.0–45.2)	36.0 (24.5–48.0)	0.50
Delivery mode, vaginal delivery [n (%)]	17 (65%)	48 (84%)	0.054
Nutrition during infancy, exclusively breastfed [n (%)]	15 (57%)	24 (42%)	0.18
Median duration from KD onset to sample collection, months (interquartile range)	11.5 (10.7–13.0)	n/a	n/a

KD, Kawasaki disease; HC, healthy control; n/a, not applicable.

**Table 2:** Clinical characteristics and laboratory indexes of patients with Kawasaki disease.

Age at the time of KD (months)	20.0 (12.7–31.7)
Conjunctival congestion [n (%)]	22 (84.6%)
Labial change [n (%)]	26 (100.0%)
Rash [n (%)]	25 (96.2%)
Lymphadenopathy [n (%)]	23 (88.5%)
Perianal exfoliation [n (%)]	17 (65.4%)
Erythema and edema of the hands and feet [n (%)]	10 (38.5%)
Desquamation of the fingers and toes [n (%)]	8 (30.8%)
Incomplete KD [n (%)]	1 (3.8%)
CRP (mg/L)	68.3 (44.4–89.5)
White blood cell (×10^9^/µL)	14.3 (11.4–17.5)
Neutrophil (%)	64.9 (55.3–74.5)
Hemoglobin (g/L)	113 (104–117)
Platelets (×10^9^/µL)	30.1 (24.7–36.9)
Sodium (mmol/L)	135 (133–136)
Albumin (g/L)	40 (35–41)
ALT (U/L)	28 (16–86)
AST (U/L)	39 (28–89)
Total bilirubin (µmol/L)	10.3 (8.6–15.4)
NT-proBNP (pg/mL)	517 (255–1309)
Treatment of acute KD	
Aspirin [n (%)]	26 (100.0%)
IVIG as initial treatment [n (%)]	15 (57.6%)
IVIG plus steroid as initial treatment [n (%)]	11 (42.3%)
IVIG as additional therapy [n (%)]	1 (3.8%)
Steroid as additional therapy [n (%)]	0 (0%)
IFX as additional therapy [n (%)]	0 (0%)
Antibiotics at the time of KD diagnosis [n (%)]	5 (19.2%)
IVIG non-response [n (%)]	11 (42.3%)
Coronary artery lesions [n (%)]	0 (0%)

Data are expressed as numbers (%) or medians (interquartile range). ALT, alanine transaminase; AST, aspartate transferase; CRP, C-reactive protein; IFX, infliximab; IVIG, intravenous immune globulin; KD, Kawasaki disease; NT-proBNP, N-terminal pro-B-type natriuretic peptide.

### 3.2 Alpha and beta diversity

**Figure 1:**
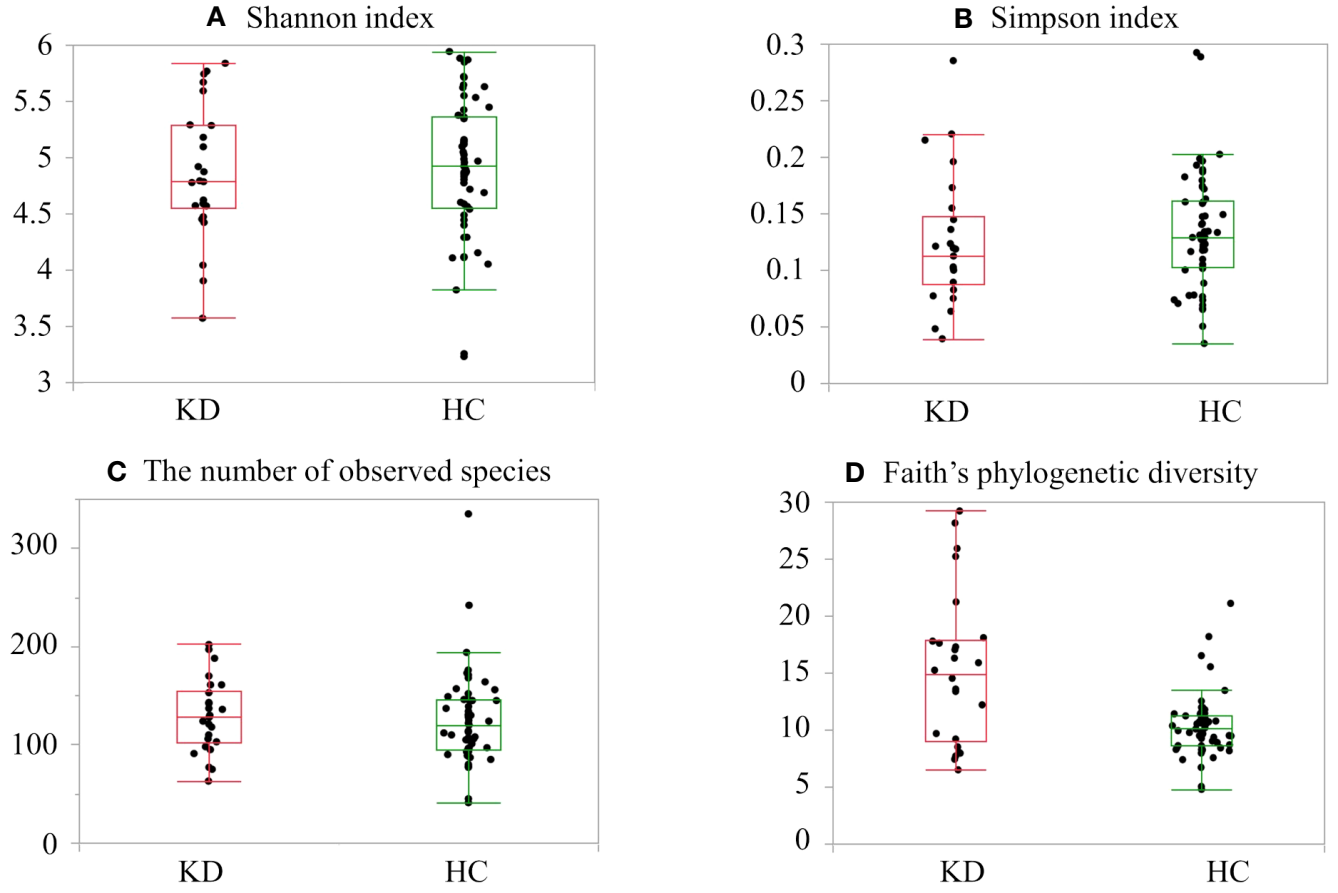
Alpha diversity in the healthy control (HC) and Kawasaki disease (KD) groups: **(A)** Shannon index, **(B)** Simpson index, **(C)** number of observed species, and **(D)** Faith’s phylogenetic diversity. The top and bottom edges of the boxes represent the 25th and 75th percentiles, respectively. Central vertical lines extend to the maximum and minimum values without outliers.

**Figure 2:**
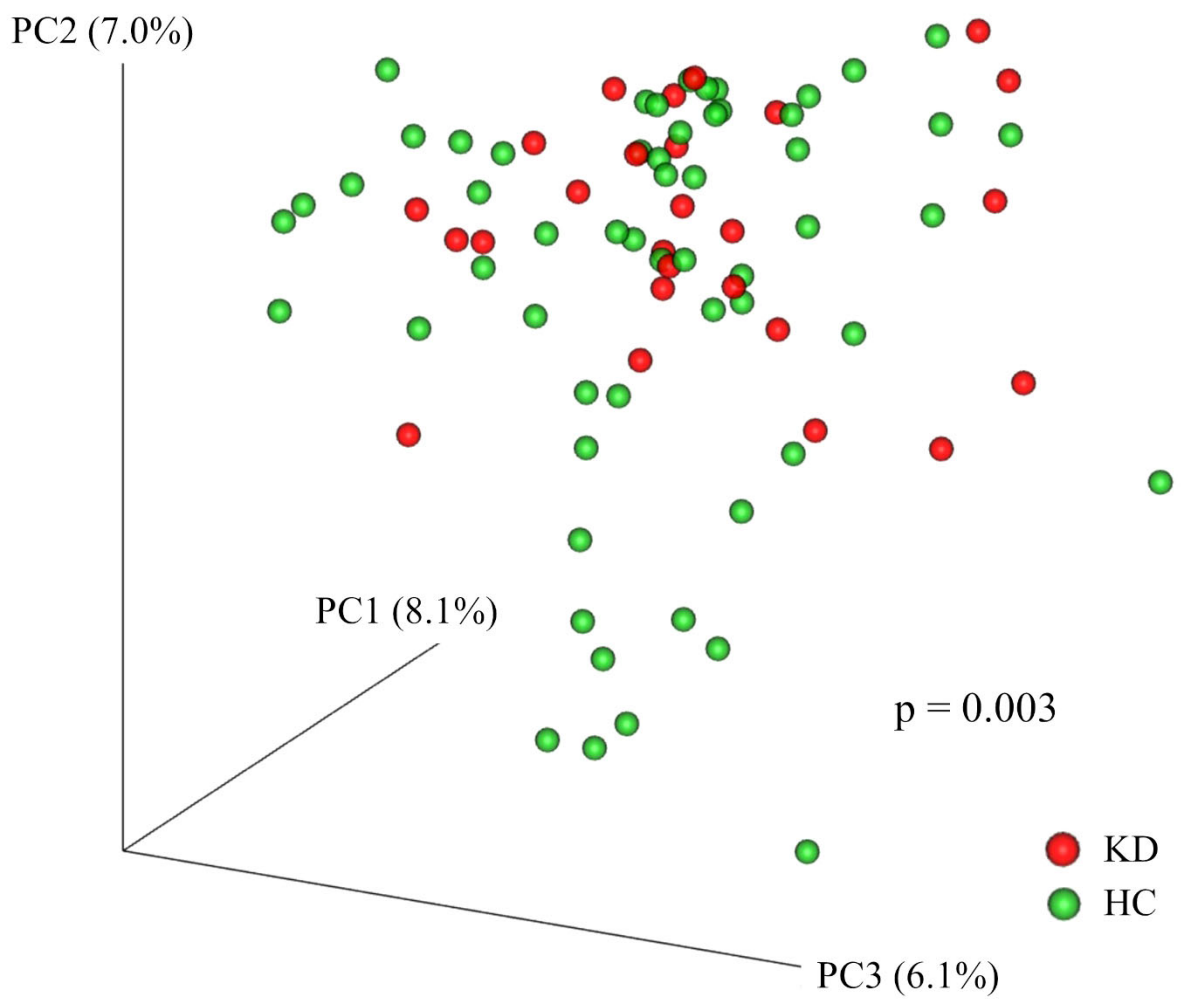
Principal coordinates analysis plot of Bray–Curtis dissimilarity. Each point represents a sample. Red points represent participants in the Kawasaki disease (KD) group. Green points represent participants in the healthy control (HC) group. The two groups formed significantly different clusters according to the permutational analysis of variance (PERMANOVA) test (p=0.003).

The KD and HC groups did not differ significantly in the Shannon index (KD group, median 4.78 (IQR, 4.54–5.28) *vs*. HC 4.92 (IQR, 4.55–5.36); p=0.64), Simpson index (0.11 (IQR, 0.08–0.14) *vs*. 0.12 (IQR, 0.10–0.16); p=0.20), or number of observed species (129 (IQR, 101–155) *vs*. 120 (IQR, 95–145); p=0.38). There were significant differences in Faith’s phylogenetic diversity (KD 14.8 (IQR, 9.0–26.5) *vs*. HC 10.0 (IQR, 8.6–11.2); p=0.005) (**Figure 1**). When comparing beta diversity based on Bray–Curtis dissimilarity, the two groups formed significantly different clusters according to the permutational analysis of variance (PERMANOVA) test (p=0.003) (**Figure 2**).

### 3.3 Microbial composition of gut microbiota

**Figure 3:**
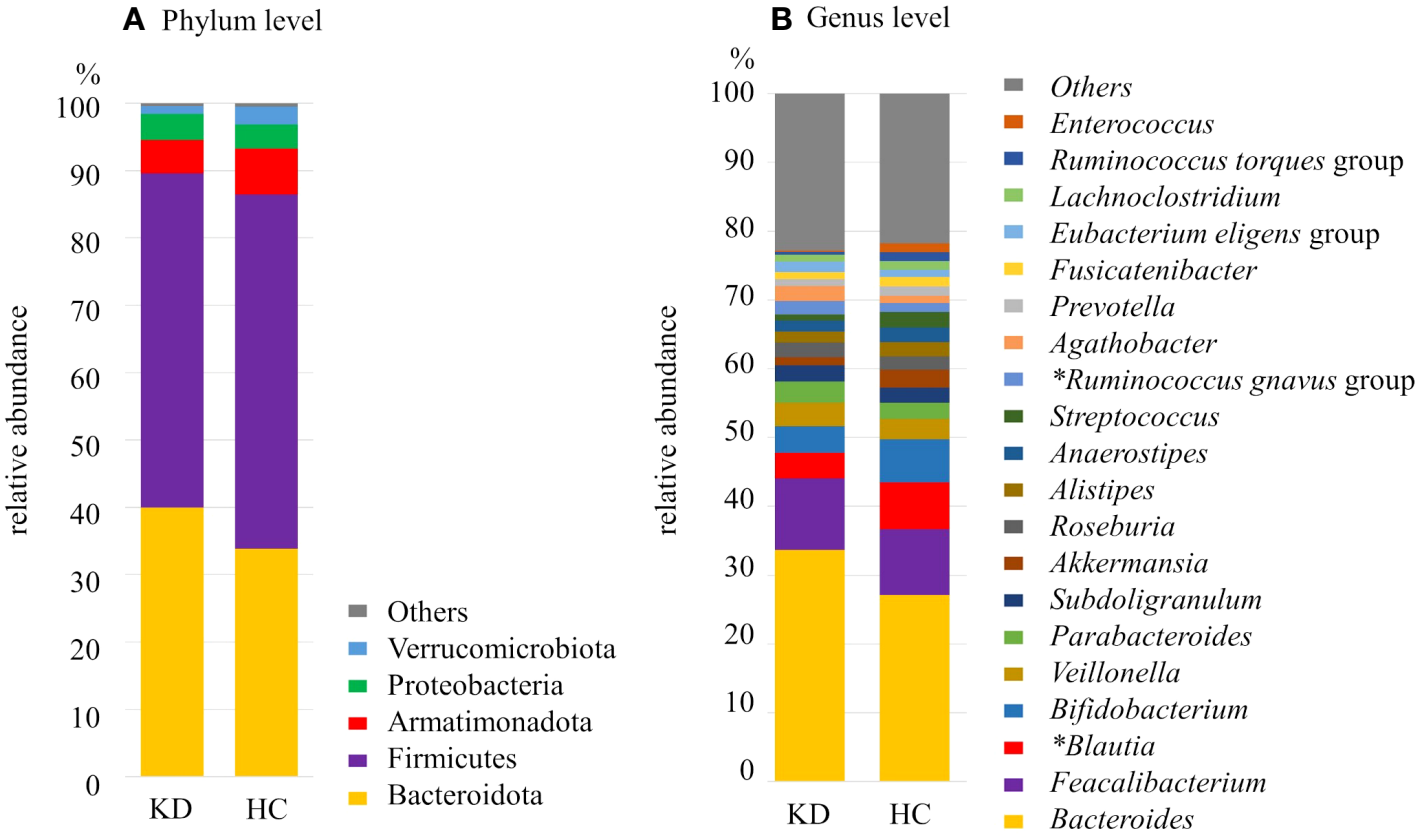
Composition of the gut microbiota of the Kawasaki disease (KD) and healthy control (HC) groups at the **(A)** phylum level and **(B)** genus level. *significant difference with false discovery rate q<0.10. The proportion (relative abundance, %) of *Blautia* was significantly higher in the HC group whereas the proportion of *Ruminococcus gnavus* group was significantly higher in the KD group.

At the phylum level, the most 5 abundant phyla observed among all samples were Firmicutes (50.1%), Bacteroidota (35.7%), Armatimonadota (6.2%), Proteobacteria (3.5%), and Verrucomicrobiota (2.1%). Regarding the relative abundance of phyla, in the KD group, Bacteroidota was more abundant (KD *vs*. HC, 39.9% *vs*. 33.8%; p=0.093) and Firmicutes and Armatimonadota were less abundant (KD *vs*. HC, 49.9% *vs*. 52.5%; p=0.42 and 4.9% *vs*. 6.8%; p=0.22, respectively) although they did not reach statistical significance (**Figure 3A**).

**Figure 4:**
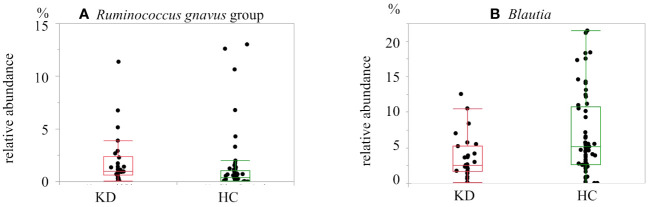
Box-and-whisker plot of the relative abundance of bacteria in the Kawasaki disease (KD) and healthy control (HC) groups: **(A)** *Ruminococcus gnavus* group and **(B)** *Blautia*. *Ruminococcus gnavus* group was more abundant in the KD group. *Blautia* was more abundant in the HC group.

At the genus level, among all samples, the dominant genus was *Bacteroides* (29.1%), followed by *Faecalibacterium* (9.8%), *Blautia* (5.8%), and *Bifidobacterium* (5.5%). Each of the other genera made up less than 5% of the samples. Twenty genera of bacteria were found in abundance greater than 1%. Among them, there were two genera that differed between the two groups after multiple comparison correction: *Ruminococcus gnavus* group was more dominant in the KD group (KD *vs*. HC, 1.9% *vs*. 1.3%; p=0.0014, q=0.028). On the other hand, *Blautia* was more abundant in the HC group (KD *vs*. HC, 3.6% *vs*. 6.8%; p=0.0094, q=0.094) (**Figures 3B**, **4**).

We also examined the associations between these genera and treatment responsiveness in the KD group (n=26). There were no significant differences in the relative abundance in any bacterial taxa that comprised of more than 1% between responders (n=15) and non-responders (n=11) to initial IVIG treatment.

### 3.4 Prediction of gut microbiota function

**Figure 5:**
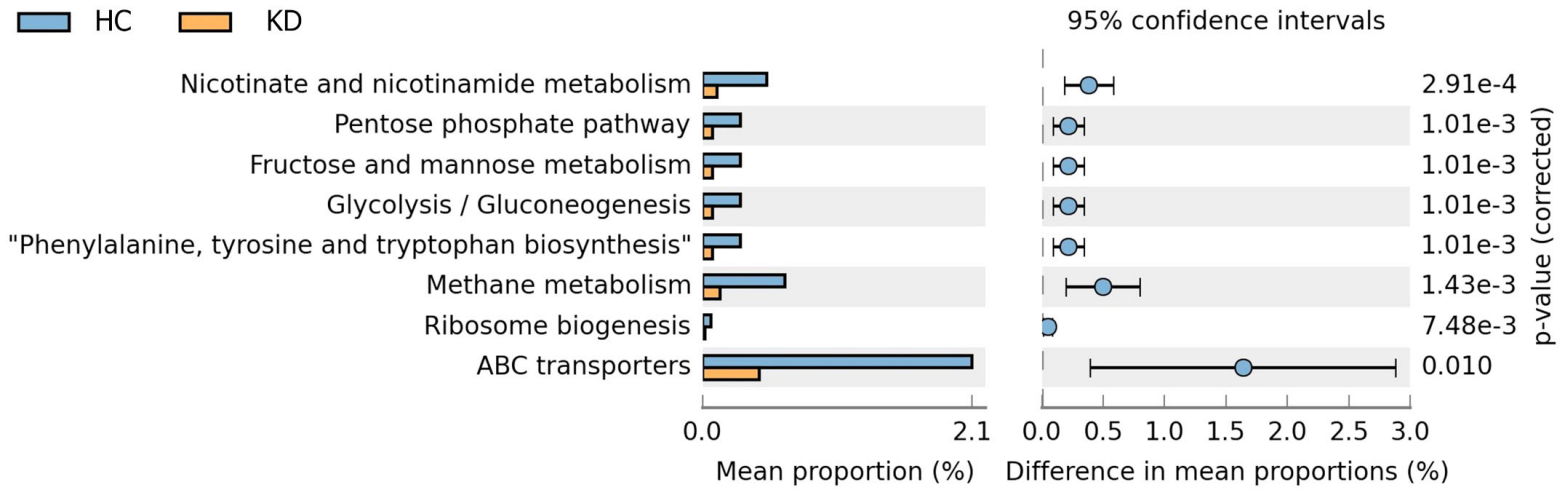
Predicted function of gut microbiota in the two groups based on Phylogenetic Investigation of Communities by Reconstruction of Unobserved States 2 (PICRUSt2) analysis of 16s rRNA data. There were significant differences in mean proportions of eight metabolic function pathways between the Kawasaki disease (KD) and healthy control (HC) groups (p<0.05). Of note, the mean proportion of bacteria involved in ATP-binding cassette (ABC) transport function was higher in healthy controls than in children with a history of KD.

Eight metabolic function pathways were identified based on KEGG. The gut microbiota of the HC group had more bacteria involved in nicotinate and nicotinamide metabolism; the pentose phosphate pathway; fructose and mannose metabolism; glycolysis and gluconeogenesis; phenylalanine, tyrosine and tryptophan biosynthesis; methane metabolism; ribosome biogenesis; and ATP-binding cassette (ABC) transport function than that of the KD group. Of note, there was a large difference in the proportion of bacteria involved in ABC transport function between the two groups (**Figure 5**). The proportion of bacteria involved in ABC transport function was higher in the HC group than in the KD group (mean proportion: KD, 2.08%; HC, 0.45%; p=0.010).

### 3.5 *Post hoc* power analysis

A *post hoc* power analysis was conducted to evaluate the relative abundance of *Blautia*. The *post hoc* power analysis yielded an effect size of 0.725 and a power of 0.907, with a type I error rate of 0.05 and sample sizes of 26 and 57 for the KD and HC groups, respectively. Power exceeded 0.8, which was set based on *a priori* power analysis.

## 4 Discussion

Several studies have reported dysbiosis in patients with KD. (7–9) However, all studies were conducted during the acute phase of KD, making it difficult to determine whether dysbiosis was caused by KD or represented a predisposition of the intestinal microbiota that existed prior to KD onset.

In order to attenuate the impacts of administered drugs and persistent fever during the acute phase of KD on the gut microbiota, we compared the gut microbiota of children with a history of KD approximately 1 year prior (non-acute phase of KD) and healthy controls in this study. As a result, we found that children with a history of KD demonstrated distinct diversity in gut microbiota: Both the alpha diversity assessed by Faith’s phylogenetic diversity and the beta diversity assessed by Bray–Curtis dissimilarity differed significantly between the groups. Considering that alpha diversity is the variation within a single sample and is used to describe compositional complexity, and beta diversity means the taxonomical differences between samples, our results suggest that children with a history of KD have characteristic gut microbiota with complexity. In addition, we also demonstrated that *Ruminococcus gnavus* group comprised more abundant in the gut microbiota, while the genus *Blautia* was less abundant in children with a history of KD.

*Blautia* produces butyrate, a short-chain fatty acid (SCFA), in the intestinal tract. Butyrate is mainly used as an energy source by colonic epithelial cells, but it is also known to induce differentiation of regulatory T cells (Tregs) in the intestinal immune system. (22) Since Tregs suppress excessive immune responses, it is possible that decreased abundance of *Blautia* suppresses Treg differentiation and increases susceptibility to KD. Indeed, a study involving a mouse model of KD showed that increasing the proportion of bacteria that produce SCFAs such as butyrate resulted in lower levels of intestinal permeability markers and inflammatory cytokines as well as fewer coronary artery aneurysms (23). Furthermore, it has been reported that *Blautia* levels are low in patients with allergic diseases with pathogenesis linked to Treg function (24) such as asthma (25, 26), atopic dermatitis (27), and chronic urticaria (28). Decreased *Blautia* levels have also been reported in the gut microbiota of patients with IgA vasculitis, (29, 30) which is the second commonest systemic vasculitis in children after KD in Japan. (31) Taken together, the lower proportion of *Blautia* in the gut microbiota might be a characteristic feature of patients with a history of KD.

In addition, another feature of the gut microbial composition of children with a history of KD was the significant abundance of *Ruminococcus gnavus* group. *Ruminococcus gnavus* group produces a pro-inflammatory polysaccharide. (32) De Filippis et al. reported that the gut microbiome of children affected by food or respiratory allergies is characterized by a higher abundance of the *Ruminococcus gnavus* group (33).

We also found differences in the function of gut microbiota between the two groups based on predictive functional analysis with PICRUSt2 and KEGG. In particular, there was a decrease in the proportion of bacteria involved in ABC transport function in the KD group. Some ABC transporters are responsible for the adhesion and settlement of lactic acid-producing bacteria and other beneficial bacteria to the mucin layer of the intestinal epithelium (34). In children with a history of KD, it is possible that fixation of beneficial bacteria is suppressed. As a result, aberrant intestinal immune system might develop.

Epidemiological studies in Japan have shown an increase in the number of cases of KD despite a decrease in the number of births. (35) Similar to the incidence of KD, the incidence of allergic and autoimmune diseases is increasing worldwide. (36, 37) In a review by Lee et al., children with allergic rhinitis, asthma, atopic dermatitis, or urticaria have a higher incidence of KD and children with a history of KD have a higher prevalence of allergic disease. (38) In fact, imbalances between T helper cell 1 (Th1) and T helper cell 2 (Th2) immune responses characterized by a skewed Th2 response have been proposed in the pathogenesis of KD (39–41) as well as in allergic diseases (42). Considering that the gut microbiome has been shown to be important in the development of either effector or tolerant responses to different antigens via the balance of Th1 and Th2 activity, (43) dysbiosis in the gut microbiome might lead to an immune imbalance with Th2 predominance, resulting in elevated susceptibility to KD as well as allergic diseases.

Regarding environmental factors that might cause gut dysbiosis, Cesarean delivery, formula feeding, and antimicrobial use during early infancy have been reported. Interestingly, Cesarean delivery (5), formula feeding (6), and history of antimicrobial use prior to the onset of KD (5) have been reported to be environmental risk factors for the development of KD. Of note, Cesarean delivery tended to be more common in children with a history of KD than in HCs in the present study, even though this difference was not statistically significant due to the small sample size.

Based on these findings, we hypothesize that various environmental factors during infancy lead to dysbiosis characterized by distinct microbial diversity and decreased abundance of *Blautia* in parallel with increased abundance of *Ruminococcus gnavus* group and that this dysbiosis might be a susceptibility factor for KD via aberrant immune responses.

There are several limitations to this study. The first major limitation is that this study was based on 16S rRNA analysis. To identify mechanisms through which the intestinal microbiota is involved in the pathogenesis of KD, it is preferable to conduct functional and metagenomic analysis. Second, the study involved children at 6–15 months after the onset of KD. Although the samples were collected after the acute phase of KD, the possibility that drugs used to treat KD, such as immunoglobulins, aspirin, steroids, and antibiotics, might have affected the intestinal microbiota cannot be ruled out. However, considering that apparent dysbiosis caused by antibiotics resolves within 3 months, (44) the effect of drugs used to treat KD might be limited after approximately 1 year. Third, we did not follow the HC group for a long period of time. Therefore, it is possible that there are potential patients with KD in the HC group. However, this possibility is unlikely because the median age at sample collection in the HC group was 36.0 months (IQR, 24.5–48.0 months) and the incidence of KD is highest among children aged 6 to 11 months. (45) Finally, we recruited participants from only one region in Japan; thus, our findings might not be generalizable to other areas.

In conclusion, the gut microbiota of children with a history of KD exhibits dysbiosis characterized by distinct microbial diversity and decreased abundance of *Blautia* in parallel with increased abundance of *Ruminococcus gnavus* group. Therefore, dysbiosis might be a susceptibility factor for KD.

## Data availability statement

All the sequence data presented in the study are deposited in DNA Data Bank of Japan Sequence Read Archive, accession number PRJDB16806. Other data sets generated during and/or analyzed during the current study are not publicly available due to privacy restrictions but are available from the Kansai Medical University Research Data Storage via the corresponding author upon reasonable request.

## Ethics statement

The studies involving humans were approved by the ethics committees of Kansai Medical University (approval no. 2015127) and Osaka Asahi Children’s Hospital (approval no. 43). The studies were conducted in accordance with the local legislation and institutional requirements. Written informed consent for participation in this study was provided by the participants’ legal guardians/next of kin.

## Author contributions

YT: Writing – original draft, Data curation, Funding acquisition, Visualization. SA: Conceptualization, Formal Analysis, Funding acquisition, Methodology, Visualization, Writing – original draft. S-IH: Writing – review & editing, Data curation. ST: Writing – review & editing, Formal Analysis, Methodology, Visualization. KH: Writing – review & editing, Formal Analysis, Methodology. KK: Conceptualization, Supervision, Writing – review & editing.
